# Low birth weight in Angola and its socioeconomic determinants

**DOI:** 10.1093/eurpub/ckac131.470

**Published:** 2022-10-25

**Authors:** J da Silva Maia, JP Teixeira, G Queiroz, J Félix China

**Affiliations:** 1 Public Health Unit, Primary Healthcare Cluster Estuário do Tejo, Lisbon, Portugal; 2 Public Health Unit, Primary Healthcare Cluster Sintra, Lisbon, Portugal; 3 Public Health Unit, Primary Healthcare Cluster Baixo Vouga, Aveiro, Portugal; 4 Antropology and Health Research Center, University of Coimbra, Coimbra, Portugal; 5 Public Health Unit, Primary Healthcare Cluster Arrábida, Setúbal, Portugal

## Abstract

**Background:**

In low-income nations, low birth weight (LBW) is still a major public health concern, which accounts for 96.5 % of global LBW cases. Any newborn weighing less than 2500g is considered LBW, which is associated with a 20-fold increase in the chance of dying during infancy. Despite the severity of the problem, in Sub-Saharan Africa the high rates of LBW have not diminished in the recent decade. The goal of this study is to evaluate the prevalence of LBW and its risk factors in Angola, as there is a pressing need to address LBW and its substantial health and social implications.

**Methods:**

We used secondary data from the Angola Children’s Record Database of Demographic and Health Survey from 2015 to 2016. A binomial logistic regression model was used to investigate the prevalence of LBW and its related risk variables.

**Results:**

From the 3738 children selected, 9.2% were born with LBW. The complete lack of formal maternal education (p = 0.011; adjOR=4.56, 95%CI 1.41-14.74), the absence of maternal iron supplementation during pregnancy (p = 0.017; adjOR=1.42, 95%CI 1.07-1.89) and women living in rural areas (p = 0.016; adjOR=1.37, 95%CI 1.06-1.78) were associated with LBW.

**Conclusions:**

Education appears to have a significant impact on LBW, emphasizing the importance of addressing literacy in Public Health policies. Lack of iron supplementation and rural residence can also be used as indicators of poor health-care access. Understanding the factors informs decision-makers and should pave the way for more targeted intervention and more efficient LBW policy.

**Key messages:**

• Intervention in socioeconomic factors and health access during pregnancy might have a high impact on the LBW problem.

• Addressing literacy as a major health determinant can guide a more efficient policy making and help stakeholders target their interventions.

